# Does Water Matter? The Impact of Water Vulnerability on Corporate Financial Performance

**DOI:** 10.3390/ijerph191811272

**Published:** 2022-09-07

**Authors:** Liyuan Zheng, Ling Ye, Mengjiao Wang, Yingdi Wang, Haiwei Zhou

**Affiliations:** 1 Business School, Hohai University, Nanjing 211100, China; 2 College of Environment, Hohai University, Nanjing 210098, China; 3 School of Public Health and Nursing, Yangzhou University, Yangzhou 225012, China

**Keywords:** water vulnerability, water governance, corporate financial performance, water regulation, water investment, water risk, sustainability, SOEs, water-intensive industry

## Abstract

This study aims to understand the potential relationship between water vulnerability and corporate financial performance for listed companies in China. Studies have argued that water risk has begun to affect the sustainability of firms, but few studies have included water conditions in the research framework to examine whether and how water conditions have a direct impact on firms. In addition, studies on environment governance have emphasized the impact of government environmental regulation on firms. This study focuses on both regulation and government investments that have been previously neglected. Using a sample of Chinese listed companies from 2016 to 2020, this paper uses pooled cross-sectional regressions with year and industry fixed effects to examine the effects of water vulnerability on corporate financial performance and analyze the mechanism of government water governance (which can be divided into water regulation and water investment) on the relationship between water vulnerability and corporate financial performance. This study finds that water vulnerability could negatively impact corporate financial performance, and water regulation can intensify but water investment couldn’t significantly relieve the negative impact. The relationships above differ between SOEs and non-SOEs and water-intensive and non-water-intensive industries.

## 1. Introduction

Water resources are one of the 17 UN Sustainable Development Goals. Global demand for clean water is expected to exceed 40% of the available supply by 2030, but the quality and quantity of freshwater have declined significantly over the last few decades due to global population growth and climate change [1]. The global water crisis has become the world’s third-biggest threat and one of the main risks that the business sector needs to address [2]. Some water intensive global giants have taken note of the problem and have begun to address the impact of water vulnerability on corporate sustainability. For example, BHP Billiton incorporates water management into its core concerns in exchange for the smooth implementation of its local mining business [3]. Nestle has taken the initiative to help farmers improve agricultural irrigation, so as to ensure the supply of agricultural materials and demonstrate its social responsibility to acquire social licenses to locally operate [4]. Studies have shown that water systems are now increasingly vulnerable and thus companies face serious physical, reputational, and regulatory water risks [5,6]. When companies internalize these risks, they already have significant implications for their business development [7,8,9]. However, most companies are still unaware of the risk and opportunities presented by water issues [10].

Despite corporations facing serious physical, reputational, and regulatory water risks simultaneously [5,6], business studies have mainly focused on reputational and regulatory risks by exploring the impact of social factors such as institutions and stakeholders under socio-centric theories and ignoring the possible direct impact of natural resources and the environment itself on business development [11,12,13,14], i.e., most scholars only treat natural resources and the environment as a scenario for research rather than incorporating them directly into their research frameworks. Although research concerning climate change issues has recently begun to focus on physical risk by exploring the direct impact of the natural environment on business financial outcomes (i.e., research from [15,16]), such research is still in its infancy and water issues are absent. The results from research considering the impact of climate change issues on firms may only have limited reference to water issues because environmental issues vary considerably [17] in terms of issue scale and stakeholder pressures [13,14,18,19,20,21].

To avoid the tragedy of the commons, government water governance is regarded as an important method to solve water issues. Apart from regulation instruments which bring pressure to urge social agents to save water resources and protect water environments, governments simultaneously invest huge funds into water infrastructure to secure water supply and retore water environments that could relieve regional physical water risk. However, current research mainly focuses on the regulation and ignores other tools [22,23,24]. Studies only from the perspective of environmental regulation do not fully capture the impact of government environmental governance, ignore the possible crowding out or multiplier effects between different instruments, and tend to lead governments to adopt more stringent regulatory tools. Overly stringent environmental regulation, while beneficial to the achievement of regulatory objectives, may negatively affect firms’ production and operations due to excessive pressure [25]. At the micro-level, this can result in companies not being able to effectively adapt to the external institutional environment in the context of sustainable development, thus hindering the achievement of corporate goals; and at the macro level, it can lead to a lack of effective micro-level pathways to the sustainability of water resources.

Thus, this paper uses pooled cross-sectional regressions with year and industry fixed effects to examine the effects of water vulnerability on corporate financial performance and analyze the moderating effect of government water governance (which can be divided into water regulation and water investment). This study finds that water vulnerability could negatively impact corporate financial performance, and water regulation can intensify but water investment couldn’t significantly relieve the negative impact in general. However, the relationships above differ between SOEs and non-SOEs and water-intensive and non-water-intensive industries. For SOEs, they seem more vulnerable to water risk and water regulation could exert its positive moderating effect on the negative relationship between water vulnerability and corporate financial performance when compared with non-SOEs. For non-water intensive industries, they suffer more losses brought by physical water risk, and water regulation could make them worse. Water investment, though having insignificant moderating effect in the full-sample regression, could worsen the effect of water vulnerability on corporate financial performance in water intensive industries.

The contribution of this paper is threefold: firstly, different environmental issues have different impacts on firms, but previous studies have mostly focused on the impact of climate change and air quality. This paper focuses on water resources, further enriching the research on the impact of natural resources and the environment on firms. Secondly, previous research on water vulnerability has focused on its measurement and its relationship with economic development, but few studies have examined the micro-level impacts of water vulnerability. This paper focuses on the impact of water vulnerability on corporate financial performance, enriching the research on water management. Thirdly, previous studies have focused on regulation, neglecting government investment in environmental issues. This paper further divides environmental governance applied by the government into two types of tools, regulation and investment, verifying that different government environmental governance tools have differential impacts, and enriching the research on environmental governance.

## 2. Literature Review

### 2.1. The Relationship between Water Resources and Economic Development

Studies suggest that water could restrain economic development through hydro-economic links if water decouples with economic development. For example, water supply disruption [26] and water-related public health [27] could raise direct economic losses. Through resource nexus, water could exacerbate conflicts among different users, thus further dampening economic development [28,29]. For example, Wang et al. [30] found that water resources are highly related to low carbon economic development. Therefore, hydrologists and economists use various hydro-economic models to seek the optimal exploitation and utilization scale of water resources to maximize production values or to minimize the costs of the reduced water supply or pollution [31,32].

The above studies mainly focus on the macro-level outcome of water conditions. The fact that water resources can limit economic development means that water resources can also have a very important impact on enterprises as micro-entities in the econom, yet existing studies rarely include water conditions and enterprise development in the same analytical framework.

### 2.2. The Impact of Nature on Firms

The environment faced by humans is composed of the natural environment (ecological sub-system) and the social environment (social sub-system). The ecological sub-system expands from the initial natural resources to include natural resources and the natural environment [33], while the social sub-system includes the socio-political-economic context, governance systems, and actors [34]. Social-ecological systems (SES) theory assumes that ecological and social sub-systems do not exist in isolation from each other but interact to have a significant impact on the system as a whole [35]. However, the socio-ecological systems theory does not require the researcher to focus on the system as a whole but rather needs to specify the level and object of the research, i.e., what subsystems, what institutions, what types of actors, etc. the researcher is focusing on [34]. In studies that address the firm as an actor, scholars have long focused on the impact of socio-environmental factors, falling into a socio-centric perspective and neglecting the direct impact that natural resources and the environment (the ecosystem subsystem) itself may have on firm development [11,12,13,14].

Attention to the interactions between ecosystems and social systems has gradually emerged in recent years, but most studies have focused on the environment and macroeconomic and political factors, such as environmental and economic development [36] and pollution and environmental regulation [22,37]. A small number of scholars also analyze data from the perspective of the firm as an actor, i.e., they study the impact of natural resources and the environment on firm development. These studies have concluded that the deterioration of the natural environment significantly increases the labor costs of firms, as a harsh natural environment not only makes the region lose its talent attractiveness to a certain extent [36] but also accelerates the brain drain from the region and increases the cost of acquiring highly qualified talent for firms [38]. Exposure of employees to the harsh natural environment increases the risk of illness [39], which also becomes a hidden employment cost for companies. Secondly, the natural environment not only reduces the productivity of a company by affecting the productive mood and work behavior of its employees [40,41] but also affects its business efficiency by influencing its trade transactions [42]. The deterioration of the natural environment also increases the likelihood that companies will experience environmental disasters, thereby increasing the risk of damage to equipment, supply chains, and production [15,43,44].

Furthermore, firms face more pressure of environmental regulation in places with harsh natural environments [37]. However, environmental regulation does not always promote business development and may also inhibit business performance to some extent [45,46]. For one, firms face pressure to transform their production, which requires them to accelerate the retirement of existing equipment and technology and to make adjustments to existing inventories that do not meet new environmental requirements. Secondly, environmental regulation may direct input factors away from the production sector and towards the pollution control sector, driving up the marginal costs of firms [47]. Firms also pay high environmental taxes and face high environmental penalties. The internalization of these environmental costs increases firms’ production costs [48]. Third, although many scholars have tested Porter’s hypothesis that the benefits of technological innovation can offset the problem of rising production costs and expenses caused by environmental regulation, innovation itself is a risky and unknown matter that does not necessarily reduce the risks faced by firms in the short term [49] and does not necessarily create enough opportunities for firms to compensate for the costs brought by environmental regulations. As a result, companies may also choose to scale down production based on cost–benefit considerations [25]. Apart from government, institutional pressure is also exerted by the communities in which companies operate. As local natural resources and the environment are part of the commons, environmental damage by firms may trigger community resistance and thus firms lose the ‘legitimacy’ to continue operating locally [50,51,52,53].

In addition, many scholars argue that the key reality of natural resource scarcity should be reaffirmed and valued for its impact on business sustainability [12,54]. The price, quantity, and quality of natural resources available are crucial to the day-to-day operations of businesses [55]. Degradation of ecosystems reduces the reliability of natural resource supplies, thereby increasing the environmental uncertainty faced by firms [13,14]. As the supply of natural resources is not in the hands of external organizations and firms do not have the means to internalize the supply of natural resources through mergers and acquisitions or strategic alliances, firms may respond to natural resource scarcity in a variety of ways, but these measures all lead to increased dependence on natural resources in the short term [14].

## 3. Hypothesis

Firstly, water vulnerability is expressed as an imbalance between the supply and demand of water resources within a region over a certain time. Companies can only obtain water from external sources, such as permits for abstraction or from water supply companies. Both government administrative permits and water company supplies correspond to the availability of local water resources [51]. The lower the availability of water, the more likely it is that the local area is at risk of water scarcity even in cases of extreme scarcity. Under such circumstances, water will be prioritized for basic social needs [56,57], while the production needs of businesses will be a lower priority, meaning that businesses may face water outages or that the water supplied will not be sufficient for daily operations. Even if water resources are relatively abundant where a company operates, the risk of water scarcity can spread downstream through the supply chain [58,59]. On the other hand, the less water available, the higher the cost of water for businesses, in terms of water prices, water rights fees, water taxes, etc., thus increasing the cost of doing business [60,61]. Furthermore, deteriorating water quality further contributes to water scarcity. Businesses also need to treat water resources etc. when using water, which increases the cost of water use and faces higher water costs when using water from the public network [61]. Deteriorating water quality also represents a deterioration in the regional environment, which can have a serious and long-term negative impact on business operations by affecting the ability of companies to raise capital and access resources such as high-quality human resources.

Secondly, water vulnerability is also manifested by changes in precipitation patterns due to climate change and an increase in the number of extreme meteorological events, resulting in changes in the amount of water available in the region in time and space scales [62,63]. The unpredictability of physical risks such as droughts and floods and major disasters can pose a serious threat to business operations [64,65]. For example, droughts can exacerbate water scarcity problems faced by firms, while floods can cause production disruptions and equipment losses [66].

Therefore, we assume that water vulnerability deteriorates the business performance of a firm and increases the business risk of the firm.

**Hypothesis** **1.** *The higher the water vulnerability, the lower the corporate financial performance*.

Water vulnerability is a measure of the state of water resource systems that are subject to shocks from human activities and natural changes, and the degree to which they are difficult to restore to their original state after a shock [67]. According to Xia Jun et al. [68], adaptive management of water resources is needed to cope with the impacts of climate change and human activities on the spatial and temporal distribution of water resources. As a public resource, government intervention is seen as an important method to ensure that public goods are allocated appropriately for social welfare to prevent the tragedy of the commons caused by the ‘free-riding’ behavior of various users [69,70]. Governments intervene mainly through regulatory instruments such as legislation and enforcement, as well as an investment such as engineering and ecological restoration [71]. There are differences in the mechanisms and effects of different policy instruments on water governance, but there is currently a focus on regulatory instruments, with little research focusing on the impact of government investment [22,23,24].

Environmental regulations are common tools applied by the government that are coercive to businesses. The stronger the pressure for environmental regulation, the greater the pressure felt by companies to maintain environmental legitimacy. Corporate environmental legitimacy means that companies demonstrate good environmental performance in the way that stakeholders expect. In the case of water resources specifically, this is expressed in terms of the expectation that companies conserve and sustainably use water resources [5,72,73]. Businesses facing regulation on water issues can feel pressure for transformation. On the one hand, companies need to accelerate the obsolescence of existing equipment and technologies and make adjustments to existing inventories that do not meet new environmental requirements, which affects business performance in the short term. On the other hand, process transformation and technological innovations are needed to improve water efficiency and reduce effluent emissions. However, innovation is inherently risky and unknown, in that technological innovation does not necessarily reduce the risks faced by firms in the short term [49]. Water regulation also means that firms face the possibility of paying high water taxes and higher water contamination penalties. Thus, water regulation can intensify the negative effect of water vulnerability on firms.

In addition to environmental regulation, governments will further enhance water infrastructure and restore water ecosystems to change the spatial and temporal distribution of water resources in terms of quantity and quality [74]. For example, the construction of the South–North Water Transfer Project by the Chinese government has changed the regional distribution of water resources and improved the adaptive capacity of water-scarce areas [75]. The improvement of the water environment and water ecology has improved the regional natural environment. As mentioned above, environmental degradation affects labor costs and operational efficiency. Thus, environmental improvements reduce labor costs and help improve business efficiency. Reservoirs and dikes can improve the region’s resilience to floods and droughts, reducing the risk of production losses. Investments such as reservoirs and water diversion projects increase the amount of water available to the region. Although these storage and supply projects can lift the price of water services, water price is relatively low and does not fully reflect its scarcity value in general [60,72]. The benefits of water investment may outweigh the cost that firms have to pay.

Thus, we hypothesize that:

**Hypothesis** **2a.** *Government water regulation positively moderates the relationship between water vulnerability and the corporate financial performance*.

**Hypothesis** **2b.** *Government water investment negatively moderates the impact of water resource vulnerability on corporate financial performance*.

## 4. Study Design and Data Description

### 4.1. Sampling

The years 2011 to 2015 were a period of significant transition for China’s water resources management system. The year 2011 saw the first introduction of the strictest water resources system in the Central Government’s No.1 Document and the Central Water Resources Work Conference, and in 2012 China issued the Opinions on Implementing the Strictest Water Resources Management System. The year 2014 saw General Secretary Xi Jinping put forward the policy of ‘Prioritizing water conservation, spatial balance, systematic management and two-handed efforts’. In 2015, the ‘Water Pollution Prevention and Control Action Plan’ (Water Ten) was promulgated. These policies have set a new path for water management in China. To reduce the impact of policy uncertainty during the transition period, data from 30 Chinese provinces and municipalities (excluding Tibet and Hong Kong, Macao, and Taiwan) spanning five years from 2016 to 2020 were selected to empirically screen the impact of water vulnerability on corporate financial performance. The impact of different types of government water governance practices on the relationship between water vulnerability and corporate financial performance is further assessed.

This paper excludes companies in the financial sector because the investment and financing behaviors of financial sector are significantly different from that of the non-financial sector. China has set minimum equity capital requirements for the financial sector, which affects the financial ratios of firms. The sample of firms with delisting risk is excluded because the risk characteristics of firms on delisting alert are significantly different from those of non-delisting alert firms, which in turn affects their financial performance. The first year of firms’ listing is also excluded, for financial indicators such as stock liquidity ratio and equity concentration have remarkable differences. After excluding firms with missing data, the paper ends up with a sample of 10,361 firms.

The data sources for this paper are as follows: financial data from CSMAR databases; water vulnerability data and government water investment data from China Statistical Yearbook, China Water Resources Statistical Yearbook, China Water Resources Bulletin and China Urban and Rural Construction Statistical Yearbook; government water regulation data from provincial government work reports. Stata 17.0 was used for the data processing.

### 4.2. Construction of Variables

#### 4.2.1. Water Vulnerability

Water vulnerability is a measure of the state of a water resource system that is subject to shocks from human activity and natural change, and the degree to which it is difficult to restore to its original state after a shock [67,76]. The current stage of water vulnerability assessment includes the indicator system approach, the simple index approach, and the model analysis approach. The indicator system approach is currently the most widely used method in water resources vulnerability studies. These studies have attempted to establish a system of indicators for evaluating water vulnerability in terms of several attributes such as adaptability, hazard risk, ecology, and environment. Some studies reflect vulnerability from an integrated perspective with the Drive-Pressure-State-Impact-Response (DPSIR) framework, the IPCC framework and the VSD (Vulnerability Scoping Diagram) framework. This paper refers to the study by Gui Zihan et al. [62], which considers water resources vulnerability as a function of four dimensions: exposure, sensitivity, hazard risk and resilience. The indicators of the relevant dimensions refer to Wang Liping et al. [76], Gui Zihan et al. [62], Liu Qianqian and Chen Yan [77] and Su Baoxian et al. [78]. The entropy method is applied to conduct a comprehensive evaluation. The specific indicators are shown in Table 1.

#### 4.2.2. Government Water Governance

This paper divides government water governance into water regulation and water investment. Water regulation is currently measured in two ways: one is to use water resources-related industrial emissions as a proxy variable; the other is to use the frequency or the proportion of water resources-related terms appearing in government work reports to reflect the government regulatory pressure [36,37]. This paper argues that government reports are an outline for the authority to administrate and implement decisions and resolutions by the law and is a yearly programmatic document to guide the focus of governmental work. Therefore, the proportion of water-related terms appearing in government work reports is a more comprehensive representation of the strength of government water regulation. In this paper, the following steps were taken to construct the water resources regulation indicators: firstly, the government work reports of 30 provinces from 2016 to 2020 were collected by hand; secondly, the government work reports were word-sorted; finally, the frequency of water resources-related words and their proportion of the total number of words in the government work reports were calculated. The specific words related to water resources include water environment, water resources, water pollution, water safety, water ecology, river and lake, river (lake) chiefs, water price, water use and water quality.

According to the China Water Statistics Yearbook, the main impacts of government investment on water resources include flood control, irrigation, drainage, water supply, hydropower, soil conservation and ecological restoration, institutional capacity building, early-stage work and others. The main purpose of hydropower investment is to address energy issues, and the institutional capacity building is mainly used for the day-to-day operations of the government. Thus, we exclude investment in hydropower and institutional capacity building, and use the amount of investment completed in the current year for our calculations. The ratio of government investment in water to local GDP is used as a proxy variable for government water investment.

#### 4.2.3. Control Variables

Regarding previous relevant studies examining the impact of natural resources and the environment on firms (for example, [15,16]), we control for market-to-net ratio (BM), firm size (SIZE), asset–liability ratio (LEV), stock liquidity ratio (Turnover), and equity concentration (SHR) at the firm level. The regional level control variables are total regional GDP (LGDP) and regional GDP growth rate (GGROWTH).

## 5. Results

### 5.1. Descriptive Findings

**Figure 1:**
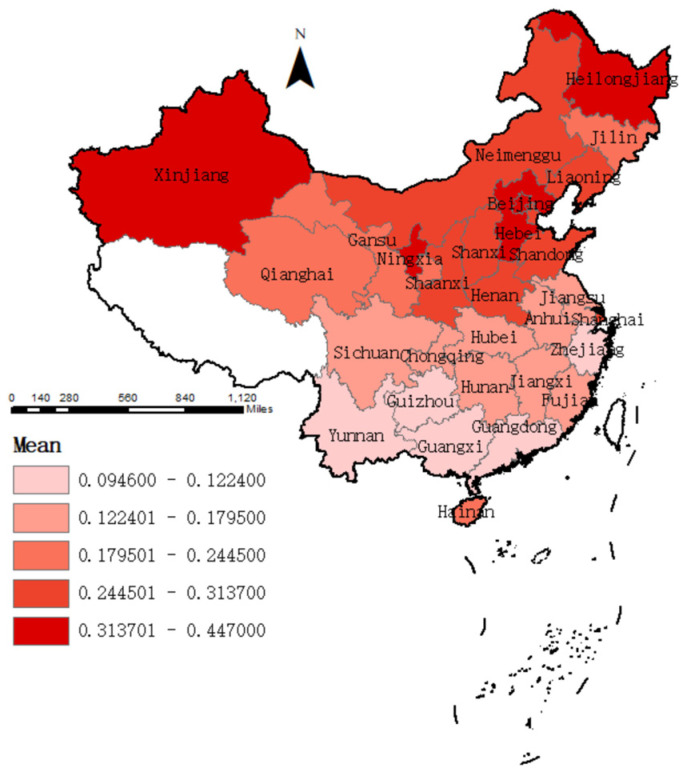
The mean level of water vulnerability of each province.

Firstly, this paper presents a descriptive analysis of water vulnerability by region and province in China. The vulnerability of water resources by region is shown in Table 2 and by province in Figure 1. The results of the descriptive statistics are generally consistent with other studies of China’s water vulnerability. Firstly, the level of water vulnerability in all regions of China is moderate and shows a decreasing trend, indicating that China has made significant progress in water resources management and has further improved its ability to ensure water security. Secondly, there are still significant regional differences in water vulnerability across China. The water vulnerability is significantly higher in the north than in the south, which is consistent with the spatial and temporal distribution of water resources in China. Particularly, water vulnerability is highest in northern China, due to the scarcity of total water resources in northern China and the high water demand of society. North China faces water resources and water environment problems such as groundwater over-exploitation and water quality deterioration [63]. Southwest China has the lowest water vulnerability, probably because it is the ‘water tower’ of China, with abundant water resources and a sparse population.

In terms of water vulnerability by province, Ningxia has the relatively highest vulnerability. Not only is Ningxia experiencing severe water shortages due to drought, but it also faces frequent natural disasters and a high spatial concentration of human activities [79]. Followed by Ningxia, Heilongjiang Province has relatively high water vulnerability, probably due to the high seasonal fluctuations in water resources, the limited water production capacity and the rapid growth in water demand from socio-economic development [80]. Provinces such as Shanghai, Beijing and Tianjin are not only under pressure from the mismatch between local water supply and demand but also face water pollution problems [81]. These provinces, therefore, have a higher water vulnerability. Provinces such as Guangdong, Guangxi, Zhejiang and Jiangxi have lower water vulnerability due to their more abundant water resources and better water environment quality. In conclusion, the spatial distribution of water resources vulnerability is generally consistent with previous studies.

To provide a more detailed analysis of how water vulnerability affects corporate financial performance, the main variables are analyzed descriptively, as shown in Table 3. Although the overall mean of ROA is not high, the difference between the very high and very low values is large, suggesting that some companies still have threats in achieving financial performance, which is not conducive to long-term future growth. Water vulnerability ranges from a maximum of 0.3918 to a minimum of 0.046, with a mean value of 0.1924, suggesting that China’s overall water vulnerability is moderate, with significant spatial variation. On the one hand, this result indicates that the Chinese government has made outstanding achievements in water governance, but on the other hand, it also suggests that companies may not be aware of water risks or underestimate the impact that water vulnerability may have. Other control variables are also considered in this paper, but the values are very similar to previous studies and are not explained here.

The Pearson correlation test (lower triangle) and the Spearman correlation test (upper triangle) were used to identify the presence of multicollinearity in the model and the results of the correlation coefficient calculations are presented in Table 4. The results show that ROA is significantly correlated with water vulnerability. This provides some indication that firms are exposed to water risk, providing partial evidence for H1. The correlation between government water governance and ROA and water vulnerability provides some support for H2. The correlation coefficients between all variables are well below 0.6, suggesting that there is no serious collinear problem.

### 5.2. Endogenity Test and Model Selection

The measurement of water vulnerability includes some indexes that are influenced by social and economic development. Despite controlling for some variables reflecting regional economic development, the variable of water vulnerability may still fail to meet the requirements of exogeneity due to the existence of omitted variables or measurement errors. To obtain more robust results, an endogeneity test is first carried out.

We follow the method presented by Wooldridge [82]. Starting with a random-effects model on the firm level, we attempt to explain corporate financial performance (ROA) using water vulnerability score (*Water_Vul*) and a set of control variables including price to book ratio (BM), total asset (SIZE), financial leverage (LEV), turnover of stocks (TOVER), share concentration (SHR), provincial GDP (LGDP) and provincial GDP growth (GGROWTH).

(1) $$ROA_{it}=\alpha_{1}+\beta_{1\;}Water\_Vul_{jt}+\sum Controls_{it}+\mu_{i}+\varepsilon_{it}\;\;\;$$

(2) $$Water\_Vul_{jt}=\alpha_{2}+\delta_{1}SUN_{jt}+\beta \delta_{2}\sum Controls_{it}+\omega_{it}$$

Then we introduce the variable of the annual hour of sunshine to test whether water vulnerability meets the requirement of endogeneity. This paper considers that water vulnerability may be affected by regional economic development levels, but the average annual hour of sunshine is less affected by social and economic development levels than water vulnerability. Firstly, we obtain the residuals from Equation (2) (see [R1] in Table 5) and include the residuals in the first Equation (1). For the corporate financial performance (ROA), the coefficient of the residual denoted *VUL_hat* exhibits a *p*-value of 0.408 (see [R2] Table 5). Thus, we accept the null hypothesis that the coefficient of the residual is equal to zero, suggesting that the water vulnerability (*Water_Vul*) is exogenous.

Despite no endogenous problem existing, some errors related to time and industries may also prevent us achieving more robust results. Thus, we adopt a pooled cross-sectional model with year fixed effect and industry fixed effect to test the relationship between water vulnerability and corporate financial performance (see Equation (3)).

(3) $$Firms_{it}=\alpha_{1}+\beta_{1\;}Water\_Vul_{jt}+\sum Controls_{it}+YEAR+INDUSTRY+\varepsilon_{it}$$

### 5.3. The Determinant of Corporate Financial Performance

Table 6 presents the test results relating to the effect of water vulnerability on corporate financial performance. The sample includes the 30 provinces listed in Table 4. R1 shows the results using the annual water vulnerability score with ROA as the dependent variable. In Column (1), the coefficient of the annual water vulnerability is −0.0282, with a 95% confidence interval of between −0.0042 and −0.0122. This indicates that moving from the first quartile (0.1135) to the third quartile (0.2132) of the water vulnerability score can reduce the financial performance of a firm by 0.26%. We find that higher water vulnerability is significantly associated with lower corporate financial performance, suggesting that water does matter to firms. H1 has been proven.

Since there are significant differences in water risk perception between water-intensive industries and non-water intensive industries, this paper further divides the samples into two sub-samples for regression. The division of water-intensive industries is not unified at present. In this paper, ten industries whose water consumption data are regulated by the National Bureau of Statistics of China are regarded as water-intensive industries, which are coal mining and washing, ferrous metal smelting and rolling processing, non-metallic mining and selection, electric power and heat production and supply, textiles, paper products industry, nonferrous metal smelting and rolling processing, chemical raw materials and chemical products manufacturing, non-metallic mineral products industry and petroleum processing, coking and nuclear fuel processing. In R2, the *p*-value of the coefficient of the annual water vulnerability is 0.209, indicating that water vulnerability cannot significantly influence the financial performance of water-intensive firms. This result seems strange and defies common sense. However, we argue that the reason why water vulnerability cannot significantly affect the financial performance of water-intensive enterprises is that these enterprises have established adaptive management of water resources to secure themselves from water vulnerability [51,83,84]. For example, Callaghan et al. [85] found that water-intensive corporations strengthened their adaptation to water vulnerability through technological innovation. Meanwhile, the coefficient still shows that water vulnerability threatens the financial performance of water-intensive enterprises.

R3 shows that the coefficient of the annual water vulnerability is −0.0314, with a 95% confidence interval of between −0.0497 and −0.0130. This indicates that moving from the first quartile (0.1135) to the third quartile (0.2132) of the water vulnerability score can reduce the financial performance of non-water-intensive enterprises by 0.313%. This implies that water risk needs more attention from non-water-intensive enterprises.

We further divide the sample into state-owned (R4) and non-state owned (R5) to test whether the ownership of enterprises buffers or intensifies the impact of water risk. Some scholars believe that state-owned enterprises can strengthen their profitability through natural resources rent [86]. However, other scholars hold the opposite opinions, in that as state-owned enterprises function as an instrument to enforce government policies, they may underperform in financial outcomes to reach non-commercial goals [87,88]. Water issues are highly related to human rights [57] (Hazelton,2013), and state-owned enterprises need to bear more responsibilities to protect civil rights. For example, Huang et al. [87] found that state-owned enterprises pay more pollutant discharge fees. Our research shows (as shown in R4 and R5) that water risk moving from the first quartile (0.1135) to the third quartile (0.2132) can reduce the financial performance of state-owned enterprises by 0.76%, while non-state-owned enterprises only suffer 0.215% losses. This demonstrates that state-owned enterprises seem more vulnerable to water risk.

### 5.4. The Impact of Government Water Regulation on the Relationship between Water Vulnerability and Corporate Financial Performance

The government applies regulation and investment tools to water governance. Table 7 shows the impact of government water regulation on the relationship between water vulnerability and corporate financial performance. In the full-sample regression, we find that government water regulation significantly intensifies the negative impact of water vulnerability on corporate financial performance (as shown in R1), indicating that the water-related risk from regulation is also non-negligible. H2a has been proven. This finding is in line with Cui and Jiang [25], Ge and Meng [45], and Long and Wan [46], and indirectly supports the opinion that corporate water management is largely promoted by institutional pressures [51,89,90,91]. R2 demonstrates that the strengthening effect of government water regulation does not appear in water-intensive industries. As discussed before, water-intensive industries have established adaptive management of water resources to secure themselves from water vulnerability [51,83,84]. Since their water use and discharge have long been monitored and regulated by the government, the effects of the increase of regulatory pressure are marginally diminishing.

When considering the difference in ownership, we find that the water regulation seems to intensify the negative effect of water vulnerability on SOE financial performance but has no significant impact on non-SOEs. As demonstrated before, SOEs may underperform in financial performance to reach non-commercial goals [87,88]. Water vulnerability threatens social welfare, so SOEs may bear more responsibility for water issues, which may increase their operating costs [87]. Secondly, departing audits of the natural environment are popular, and SOEs are more vulnerable to departing audits partly because the senior executives of SOEs in China have the status of administrative cadre [92]. However, the positive moderating effects of water regulation that do not appear in the non-SOE group may be due to the pressure of water regulation being relatively small, as many institutional ambiguities exist [93], and non-SOEs receive less attention from the public [94].

### 5.5. The Impact of Government Water Investment on the Relationship between Water Vulnerability and Corporate Financial Performance

Apart from water regulation, China’s government pays more attention to water investment to change local hydrology and water environment conditions. We assumed that these investments could somehow improve local water conditions so that corporate financial outcomes would be safeguarded. However, the finding in Table 8 shows that investment only insignificantly weakens the negative impact of water vulnerability on corporate financial performance (R1). H2b has not been proven. We interpret this as the local hydrology condition being relatively stable, and that only long-term large-scale investment in water-related infrastructures can significantly change water vulnerability. Thus, cotemporally finished water investment hardly affects cotemporal corporate financial performance.

However, the government water investment intensified the negative effect of water vulnerability on the corporate financial performance of water-intensive industries at a slightly significant level (R2). We believe this is due to those investments also needing to be paid back by water price, despite a long payback period. A small increase in water price may have little impact on non-water intensive enterprises (R3), but it has a significant impact on water-intensive enterprises.

## 6. Conclusions and Implications

Our analysis highlights a previously under-appreciated economic cost of water vulnerability. Our results suggest that companies in places with great exposure to water vulnerability exhibit higher financial losses, and water governance applied by the government can partly significantly influence the impact of water vulnerability. Specifically, water vulnerability negatively impacts corporate financial performance, and water regulation intensifies the negative impact while water investment insignificantly reduces the negative impact. Our main results are consistent with Northey et al. [51] and Ortas et al. [53] that water conditions matter to firms. However, Northey et al. [51] and Ortas et al. [53] did not investigate the impact on corporate financial outcomes. Our results are also consistent with the research made by Huang et al. [15], and Kling et al. [16] on the impact of climate risk on corporate financial results, and provide evidence related to Tashman [13] in that firms are indeed dependent on natural resources. However, Huang et al. [15], and Kling et al. [16] did not consider the impact of governance and Tashman and Rivera [14] mainly considered regulation tools. Li Zihao and Yuan Bingbing [22] considered different governance tools, but they failed to examine the different impacts on corporate financial outcomes and did not consider the effect of natural vulnerability.

Furthermore, we re-examined the relationship above in different sub-samples and found some interesting results. Water-intensive enterprises seem immune to water vulnerability, for they may have implemented water adaptative management. The effect of water regulatory pressure on them seems to diminish, while water investment may generate short-term harm because of the increase in price of water supply services. Non-water intensive enterprises are vulnerable to water vulnerability and are sensitive to regulation. This may be due to their carelessness in water adaptive management. Although the increase of water price does not bring in obvious financial losses to non-water intensive companies, their water use is anticipated to be monitored by the government. Compared with non-SOEs, SOEs suffer relatively more short-term economic loss when faced with water vulnerability due to their attributes of having more responsibilities in social welfare.

Our results have some implications. For firms, water vulnerability matters to their financial performance. Enterprises should work together to build water security; non-water intensive enterprises should especially establish the awareness of water risk and conduct water adaptive management and non-SOEs should share the responsibility for water issues. Secondly, firms should conduct water adaptive management to establish competitive advantages. Considering that the water consumption standards for several industries are under-enacted after the National Water Action Plan was issued in 2021, water institutional pressure, which is relatively low, may be intensified, especially on non-SOE businesses. For the government, water institutional pressure is needed and should be intensified because most firms do not realize the threats of water risk and the importance of water adaptive management at the firm level. However, the focus should shift to non-SOEs and non-water intensive firms by strengthening their water consumption and contaminants discharge auditing or so on. Secondly, the negative impact of water regulation on businesses also needs to be considered when increasing the pressure on water regulation in the future. Regulation tools should be used in conjunction with other governance tools. Thirdly, despite water investments not generating immediate benefits for most enterprises, they are generally beneficial in mitigating regional water vulnerability in the long run. Governments should attach more importance to this and invest in water infrastructures.

However, this study is subject to some limitations. First, although we attempted to employ as full a dataset as possible of all listed firms in the Chinese main board market, there may still be a selection bias because listed firms are a relatively small subset of all companies in China. Therefore, one should be cautious when interpreting our results for general Chinese firms. Second, although we took as many commonly used indicators to measure water vulnerability as possible, the indicators of water vulnerability are far from agreed upon. Hydrologists are still striving to explore more accurate indicators and methods to measure water vulnerability. More measurements of water vulnerability could be used to re-examine the relationship between physical water risk and firm financial outcomes. Thirdly, we mentioned that different governance tools may have multiple or cross-out effects, but we did not further test whether the multiple or cross-out effects exist in our study. Finally, we only tested the cotemporal effect. However, water vulnerability and water governance tools may have an intertemporal effect on corporate financial outcomes that deserve investigation.

## Figures and Tables

**Table 1:** The measurement of water vulnerability.

Water Vulnerability	Sensitivity	Absolute value of annual precipitation variation
		Absolute value of annual total water resources variation
	Exposure	GDP per capita
		Share of GDP in primary production
		Water resources per capita
		Total water resources/total area of the region
		Total water resources/total annual rainfall
		Proportion of high-quality surface water
		Ratio of water resources supply to demand
		Proportion of groundwater supply
		Water consumption of CNY 10,000 of GDP
		Water consumption of CNY 10,000 of industrial value added
		Water consumption by urban residents for domestic use
		Water consumption for agricultural irrigation
	Hazard	Regional population affected/National population affected in the year
		Regional direct economic losses/National losses in the year
		Area of crops affected in the region/National damage in the year
		Population with drinking water difficulties in the region/National population affected in the year
	Adaptivity	Wastewater treatment rate
		Water reuse rate
		Ratio of protected arable land
		Protection of population ratio
		Effective utilization rate of irrigation water

**Table 2:** Descriptive statistics of water vulnerability.

Region	2016	2017	2018	2019	2020	Mean
North China	0.3338	0.3270	0.2822	0.3206	0.2681	0.3063
Northeast China	0.3088	0.3146	0.3080	0.2687	0.2648	0.2930
Northeast China	0.3313	0.3105	0.3058	0.2841	0.2626	0.2989
Eastern China	0.2275	0.1817	0.1854	0.1864	0.1654	0.1893
South-Central-China	0.1876	0.1764	0.1645	0.1672	0.1487	0.1689
Southwest China	0.1411	0.1361	0.1297	0.1250	0.1165	0.1297

**Table 3:** Overall descriptive statistics.

Variables	Obs	Mean	S.D.	Min	Max
*Water_Vul*	10,361	0.1924	0.0909	0.0646	0.3918
*Water_Ins*	10,361	0.0019	0.0007	0.000	0.0049
*Water_Inv*	10,361	0.0055	0.0034	0.0013	0.0256
ROA	10,361	0.0558	0.0451	0.0018	0.2280
BM	10,361	0.6289	0.2473	0.1219	1.1891
SIZE	10,361	22.3425	1.2456	20.1137	26.1395
LEV	10,361	0.4007	0.1832	0.0638	0.8365
TOVER	10,361	5.6654	0.7380	3.6735	7.1963
SHR	10,361	0.1551	0.1070	0.0172	0.5205
LGDP	10,361	10.7181	0.6788	8.4992	11.6151
GGROWTH	10,361	5.9859	2.0171	0.5000	9.3000

**Table 4:** Correlation test.

	*Water_Vul*	*Water_Ins*	*Water_Inv*	ROA	BM	SIZE	LEV	TOVER	SHR	LGDP	GGROWTH
*Water_Vul*		−0.1642 **	−0.1033 ***	−0.1244 ***	0.0553 ***	0.0999 ***	0.0492 ***	−0.0326 ***	0.0273 ***	−0.4710 ***	−0.0795 ***
*Water_Ins*	−0.2048 ***		0.1280 ***	0.0239 **	−0.0473 ***	−0.0553 ***	−0.0215 **	0.0744 ***	−0.0212 **	0.2269 ***	0.0595 ***
*Water_Inv*	0.0552 ***	0.1705 ***		0.0001	−0.0031	0.0356 ***	0.0115	0.0393 ***	0.0121	−0.4681 ***	0.1281 ***
ROA	−0.1076 ***	0.0152	−0.0283 ***		−0.3375 ***	−0.0909 ***	−0.3819 ***	−0.1053 ***	0.1294 ***	0.1228 ***	0.0151
BM	0.0774 ***	−0.0517 ***	0.0170 ***	−0.3718 ***		0.5178 ***	0.3962 ***	−0.2727 ***	0.0872 ***	−0.0665 ***	−0.0786 ***
SIZE	0.1328 ***	−0.0514 ***	0.0401 ***	−0.0767 ***	0.5318 ***		0.5249 ***	−0.3516 ***	0.1244 ***	−0.1314 ***	−0.0364 ***
LEV	0.0573 ***	−0.0153	0.0344 ***	−0.3656 ***	0.3947 ***	0.5323 ***		−0.0506 ***	0.0260 ***	−0.0694 ***	−0.0265 ***
TOVER	−0.0457 ***	0.0724 ***	0.0404 ***	−0.0998 ***	−0.2814 ***	−0.3859 ***	−0.0603 ***		−0.3103 ***	0.0763 ***	−0.1503 ***
SHR	0.0477 ***	−0.0274 ***	0.0100	0.1043 ***	0.1149 ***	0.2067 ***	0.0518 ***	−0.3561 ***		−0.0320 ***	0.0040
LGDP	−0.4495 ***	0.1300 ***	−0.5435 ***	0.1071 ***	−0.0730 ***	−0.1277 ***	−0.0763 ***	0.0531 ***	−0.0401 ***		−0.1086 ***
GGROWTH	−0.0422 ***	0.0889 ***	0.0811 ***	0.0085	−0.0319 ***	−0.0514 ***	−0.0238 **	−0.1797 ***	0.0041	−0.0165 *	

Note: ***, **, * indicate significance at the 1%, 5% and 10% levels, respectively.

**Table 5:** Endogeneity test.

	R1	R2
		ROA
*Water_Vul*		−0.0326 ***
*VUL_hat*		−0.0081
*SUN*	0.2132 ***	
*SUN_hat*		
BM	−0.0210 ***	−0.0766 ***
SIZE	0.0061 ***	0.0108 ***
LEV	−0.0168 ***	−0.0895 ***
TOVER	0.0027 **	−0.0066 ***
SHR	0.0161 **	0.0328 ***
LGDP	−0.0350 ***	0.0044 ***
GGROWTH	0.0024 ***	−0.0004 **
Constant	−1.1754 ***	−0.1067 ***
Adj.R^2^	0.2132 ***	0.30
Obs	10,361	10,361

Note: ***, ** indicate significance at the 1% and 5%, respectively.

**Table 6:** The impact of water vulnerability on corporate financial performance.

	R1	R2	R3	R4	R5
*Water_Vul*	−0.0282 ***	−0.0210	−0.0314 ***	−0.0450 ***	−0.0216 **
BM	−0.0911 ***	−0.0819 ***	−0.0924 ***	−0.0853 ***	−0.0920 ***
SIZE	0.0128 ***	0.0136 ***	0.0123 ***	0.0153 ***	0.0138 ***
LEV	−0.0862 ***	−0.0935 ***	−0.0841 ***	−0.0806 ***	−0.0881 ***
TOVER	−0.0039 ***	0.0056 ***	−0.0070 ***	0.0015	−0.0052 ***
SHR	0.0344 ***	0.0490 ***	0.0298 ***	0.0107 **	0.0581 ***
LGDP	0.0033 ***	0.0029 **	0.0036 ***	0.0008	0.0028 ***
GGROWTH	0.0009 ***	0.0009	0.0008 *	0.0013 ***	0.0007
Constant	−0.1821 ***	−0.2725 ***	−0.1520 ***	−0.2510 ***	−0.1857 ***
YEAR EFFECT	YES	YES	YES	YES	YES
INDUSTRY EFFECT	YES	YES	YES	YES	YES
Obs	10,363	2346	8017	3001	7362

Note: ***, **, * indicate significance at the 1%, 5% and 10% levels, respectively.

**Table 7:** The moderating effect of water regulation on corporate financial performance.

	R1	R2	R3	R4	R5
*Water_Vul*	−0.028 ***	−0.0099	−0.0339 ***	−0.0374 ***	−0.0237 **
*Water_Ins*	0.0737	0.9966 **	0.2227	0.4188	0.1689
Water_Vul * Water_Ins	−5.9954 **	−4.177	−8.2806 ***	−12.4978 ***	−5.3628
BM	−0.0911 ***	−0.0815 ***	−0.0924 ***	−0.0850 ***	−0.0920 ***
SIZE	0.0128 ***	0.01352 ***	0.0122 ***	0.0151 ***	0.0138 ***
LEV	−0.0861 ***	−0.0934 ***	−0.0840 ***	−0.0806 ***	−0.0882 ***
TOVER	−0.0039 ***	0.0057 ***	−0.0069 ***	0.0016	−0.0052 ***
SHR	0.0344 ***	0.0487 ***	0.0297 ***	0.0110 **	0.0582 ***
LGDP	0.0037 ***	0.0043 ***	0.0039 ***	0.0018 *	0.0029 ***
GGROWTH	0.0008 **	0.0005	0.0008 *	0.0010 **	0.0007
Constant	−0.1862 ***	−0.2566 ***	−0.1587 ***	−0.2619 ***	−0.1865 ***
YEAR EFFECT	YES	YES	YES	YES	YES
INDUSTRY EFFECT	YES	YES	YES	YES	YES
Obs	10,363	2346	8017	3001	7362

Note: ***, **, * indicate significance at the 1%, 5% and 10% levels, respectively.

**Table 8:** The moderating effect of water investment on capital cost.

	R1	R2	R3	R4	R5
*Water_Vul*	−0.0286 ***	−0.0183	−0.0319 ***	−0.0461 ***	−0.0216 **
*Water_Inv*	0.2408	0.2245	0.2135	0.1286	0.0267
Water_Vul * Water_Inv	0.1766	−3.6891 *	1.5254	0.9123	−0.6186
BM	−0.0911 ***	−0.0815 ***	−0.0923 ***	−0.0855 ***	−0.0920 ***
SIZE	0.0128 ***	0.0135 ***	0.0123 ***	0.0154 ***	0.0138 ***
LEV	−0.0863 ***	−0.0935 ***	−0.0842 ***	−0.0810 ***	−0.0881 ***
TOVER	−0.0040 ***	0.0057 ***	−0.0070 ***	0.0014	−0.0052 ***
SHR	0.0343 ***	0.0484 ***	0.0297 ***	0.0106 **	0.0582 ***
LGDP	0.0040 ***	0.0033 *	0.0042 ***	0.0013	0.0029 **
GGROWTH	0.0007 *	0.0008	0.0006	0.0011 ***	0.0006
Constant	−0.1898 ***	−0.2562 ***	−0.1587 ***	−0.2574 ***	−0.1865 ***
YEAR EFFECT	YES	YES	YES	YES	YES
INDUSTRY EFFECT	YES	YES	YES	YES	YES
Obs	10,363	2346	8017	3001	7362

Note: ***, **, * indicate significance at the 1%, 5% and 10% levels, respectively.

## Data Availability

The data involved in the study can be obtained from the corresponding author at reasonable request.

## References

[1] CEO Water Mandate (2008). The CEO Water Mandate Transparency Framework (Phase One).

[2] World Economic Forum (2017). The Global Risks Report 2016.

[3] Calderon C.A. (2020). BHP mine water management: An integrated approach to manage risk and optimise resource value. Slope Stability 2020, Proceedings of the 2020 International Symposium on Slope Stability in Open Pit Mining and Civil Engineering, Perth, Australia, 12–14 May 2020.

[4] Sojamo S., Larson E.A. (2012). Investigating food and agribusiness corporations as global water security, management, and governance agents: The case of Nestlé, Bunge and Cargill. Water Altern..

[5] Jones P., Hillier D., Comfort D. (2015). Corporate water stewardship. J. Environ. Stud. Sci..

[6] Tello E. (2013). From Risks to Shared Value? Corporate Strategies in Building a Global Water Accounting and Disclosure Regime. Soc. Environ. Account. J..

[7] Afrin R., Peng N., Bowen F. (2021). The Wealth Effect of Corporate Water Actions: How Past Corporate Responsibility and Irresponsibility Influence Stock Market Reactions. J. Bus. Ethics.

[8] Liu C., Su K., Zhang M. (2021). Water disclosure and financial reporting quality for social. changes: Empirical evidence from China. Technol. Forecast. Soc. Change.

[9] Weber O., Saunders-Hogberg G. (2020). Corporate social responsibility, water management, and financial performance in the food and beverage industry. Corp. Soc. Responsib. Environ. Manag..

[10] Burritt R.L., Christ K.L. (2017). The need for monetary information within corporate water accounting. J. Environ. Manag..

[11] Dias C., Rodrigues R.G., Ferreira J.J. (2021). Linking natural resources and performance of. small agricultural businesses: Do entrepreneurial orientation and environmental sustainability orientation matter?. Sustain. Dev..

[12] George G., Schillebeeckx S.J., Liak T.L. (2015). The management of natural resources: An overview and research agenda. Acad. Manag. J..

[13] Tashman P. (2021). A Natural Resource Dependence Perspective of the Firm: How and Why Firms. Manage Natural Resource Scarcity. Bus. Soc..

[14] Tashman P., Rivera J. (2016). Ecological uncertainty, adaptation, and mitigation in the U.S. ski. resort industry: Managing resource dependence and institutional pressures: Ecological Uncertainty, Adaptation, and Mitigation. Strateg. Manag. J..

[15] Huang H.H., Kerstein J., Wang C. (2018). The impact of climate risk on firm performance and. financing choices: An international comparison. J. Int. Bus. Stud..

[16] Kling G., Volz U., Murinde V., Ayas S. (2021). The impact of climate vulnerability on firms’ cost of capital and access to finance. World Dev..

[17] Tan S.-H., Habibullah M.S., Tan S.-K., Choon S.-W. (2017). The impact of the dimensions of environmental performance on firm performance in travel and tourism industry. J. Environ. Manag..

[18] Bowen F.E., Bansal P., Slawinski N. (2018). Scale matters: The scale of environmental issues in corporate collective actions. Strateg. Manag. J..

[19] Craig C.A., Ma S., Karabas I., Feng S. (2021). Camping, weather, and disasters: Extending. the Construal Level Theory. J. Hosp. Tour. Manag..

[20] Whiteman G., Cooper W.H. (2011). Ecological Sensemaking. Acad. Manag. J..

[21] Perez-Batres L.A., Doh J.P., Miller V.V., Pisani M.J. (2012). Stakeholder Pressures as. Determinants of CSR Strategic Choice: Why do Firms Choose Symbolic Versus Substantive Self-Regulatory Codes of Conduct?. J. Bus. Ethics.

[22] Li Z., Yuan B. (2021). Environmental policy mechanism of local governments in the treatment of haze pollution: Policy tools, spatial correlations and threshold effects. Resour. Sci..

[23] Ban L., Liu X. (2021). The Study of Emission Reduction Effects of Different. Environmental Regulations on the Different Environmental Pollution Types. Ningxia Soc. Sci..

[24] Ma H., Hou G. (2020). Smog Pollution, Local Government Behavior and Enterprise. Innovation Intention--Based on the Empirical Data of Listed Manufacturing Companies. Soft Sci..

[25] Cui G., Jiang Y. (2019). The Influence of Environmental Regulation on the. Behavior of Enterprise Environmental Governance: Based on a Quasi-Natural Experiment of New Environmental Protection Law. Bus. Manag. J..

[26] Jun H.J., Park J.K., Bae C.H. (2020). Economic Valuation of Aging Water Main. Improvements. J. Pipeline Syst. Eng. Pract..

[27] Fuente D., Mosites E., Bressler S., Eichelberger L., Lefferts B., January G., Singleton R., Thomas T. (2022). Health-related economic benefits of universal access to piped water in Arctic communities: Estimates for the Yukon-Kuskokwim Delta region of Alaska. Int. J. Hyg. Environ. Health.

[28] Carlino A., Vita A.D., Giuliani M., Zamberletti P., Capros P., Recanati F., Kannavou M., Castelletti A. (2021). Hydroclimatic change challenges the EU planned transition to a carbon neutral electricity system. Environ. Res. Lett..

[29] Hou J.-J., Wang Z., Zhang J.-T., Yu S.-W., Liu L.-C. (2022). Revealing energy and water. hidden in Chinese regional critical carbon supply chains. Energy Policy.

[30] Wang F., Chun W., Cui Y. (2022). Urban water resources allocation and low-carbon economic. development based on soft computing. Environ. Technol. Innov..

[31] Di D., Wu Z., Wang H., Zhang F. (2022). Spatial pattern analysis on the functions of water. resources economic–social–ecological complex system. J. Clean. Prod..

[32] Dou M., Zuo Q., Ma J., Li G. (2016). Simulation and control of the linked systems of water. quantity-water quality-socio-economics in the Huaihe River basin. Hydrol. Sci. J..

[33] Bograd S.J., Kang S., Di Lorenzo E., Horii T., Katugin O.N., King J.R., Lobanov V.B., Makino M., Na G., Perry R.I. (2019). Developing a Social–Ecological–Environmental System Framework to Address Climate Change Impacts in the North Pacific. Front. Mar. Sci..

[34] McGinnis M.D., Ostrom E. (2014). Social-ecological system framework: Initial changes and. continuing challenges. Ecol. Soc..

[35] Ostrom E. (2009). A General Framework for Analyzing Sustainability of Social-Ecological. Systems. Science.

[36] Chen S.Y., Chen D.K. (2018). Air Pollution, Government Regulations and High-quality. Economic Development. Econ. Res. J..

[37] Zhuo C., Minjie P. (2018). Haze Pollution and the Strategic Choice of Local Government’s Environmental Regulation Competition. Collect. Essays Financ. Econ..

[38] Shen Y., Lu M., Zhu L., Xu G. (2021). Compensation in the smog: Air quality. and corporate executive compensation stickiness. J. Ind. Eng. Eng. Manag..

[39] Li H., Guo H., Huang N., Ye J. (2020). Health risks of exposure to waste pollution: Evidence. from Beijing. China Econ. Rev..

[40] Cai X., Lu Y., Wang J. (2018). The impact of temperature on manufacturing worker. productivity: Evidence from personnel data. J. Comp. Econ..

[41] Cao Y., Wang Q., Zhou D. (2022). Does air pollution inhibit manufacturing productivity in. Yangtze River Delta, China? Moderating effects of temperature. J. Environ. Manag..

[42] Li C., Li H. (2017). Impact of Air Pollution on Corporate Inventories—An Empirical Study. Based on Data from Manufacturing Firms in China. Manag. World.

[43] Oh C.H., Oetzel J. (2022). Multinational enterprises and natural disasters: Challenges and. opportunities for IB research. J. Int. Bus. Stud..

[44] Winn M., Kirchgeorg M., Griffiths A., Linnenluecke M.K., Günther E. (2011). Impacts from climate change on organizations: A conceptual foundation. Bus. Strategy Environ..

[45] Jingfang G., Wei S., Ting M. (2021). Influence Mechanism of Environmental Regulation. on Enterprise Profitability: Evidence from Sugar Firms in Guangxi Zhang Autonomous. Manag. Rev..

[46] Long X., Wan W. (2017). Environmental Regulation, Corporate Profit Margins and Compliance Cost Heterogeneity of Different Scale Enterprises. China Ind. Econ..

[47] Greenstone M., List J.A., Syverson C. (2012). The Effects of Environmental Regulation on the Competitiveness of U.S. Manufacturing.

[48] Ji L., Su M. (2016). The Motivation of the Environmental Costs Internalization: Is It for Policy Compliance or for Profits?—Empirical Evidence from Chinese Listed Companies in Heavy Polluting Industries. Account. Res..

[49] Yan X., Zhang Y., Pei L.-L. (2021). The impact of risk-taking level on green technology. innovation: Evidence from energy-intensive listed companies in China. J. Clean. Prod..

[50] Jia F., Hubbard M., Zhang T., Chen L. (2019). Water stewardship in agricultural supply chains. J. Clean. Prod..

[51] Northey S.A., Mudd G.M., Werner T.T., Haque N., Yellishetty M. (2019). Sustainable water. management and improved corporate reporting in mining. Water Resour. Ind..

[52] Oh C.H., Shapiro D., Ho S.S.H., Shin J. (2020). Location matters: Valuing firm-specific nonmarket risk in the global mining industry. Strateg. Manag. J..

[53] Ortas E., Burritt R.L., Christ K.L. (2019). The influence of macro factors on corporate water. management: A multi-country quantile regression approach. J. Clean. Prod..

[54] Figge F., Hahn T. (2021). Business- and environment-related drivers of firms’ return on natural resources: A configurational approach. Long Range Plan..

[55] Starik M., Rands G.P. (1995). Weaving an Integrated Web: Multilevel and Multisystem. Perspectives of Ecologically Sustainable Organizations. Acad. Manag. Rev..

[56] Appiah D.O., Abass K. (2014). Water supply and mining: The policy paradox in Ghana. Water Policy.

[57] Hazelton J. (2013). Accounting as a human right: The case of water information. Account. Audit. Account. J..

[58] Allan J.A. (2003). Virtual Water—The Water, Food, and Trade Nexus. Useful Concept or. Misleading Metaphor?. Water Int..

[59] Vos J., Hinojosa L. (2016). Virtual water trade and the contestation of hydrosocial territories. Water Int..

[60] Martinez F. (2015). A Three-Dimensional Conceptual Framework of Corporate Water. Responsibility. Organ. Environ..

[61] Fogel D.S., Palmer J.E. (2014). Water as a corporate resource. J. Glob. Responsib..

[62] Gui Z., Chen X., He Y. (2021). Spatiotemporal analysis of water resources system vulnerability. in the Lancang River Basin, China. J. Hydrol..

[63] Sun M., Kato T. (2021). Spatial-temporal analysis of urban water resource vulnerability in. China. Ecol. Indic..

[64] Bonnafous L., Lall U., Siegel J. (2017). A water risk index for portfolio exposure to climatic. extremes: Conceptualization and an application to the mining industry. Hydrol. Earth Syst. Sci..

[65] Jiao S., Li W., Wen J. (2021). Spatiotemporal changes of manufacturing firms in the flood prone Yangtze Delta. Environ. Hazards.

[66] Song Y., Ma L., Yang L., Jiang X. (2019). Business interruption risk analysis based. on fuzzy BN:a case study of flood disaster. China Saf. Sci. J..

[67] Xia J., Weng J., Chen J., Qiu B. (2014). Multi-scale Water Vulnerability Assessment Research. J. Basic Sci. Eng..

[68] Xia J., Qiu B., Li Y. (2012). Water resources vulnerability and adaptive management in the. Huang, Huai and Hai river basins of China. Water Int..

[69] Hepburn C. (2010). Environmental policy, government, and the market. Oxf. Rev. Econ. Policy.

[70] Qin Y., Harrison J., Chen L. (2019). A framework for the practice of corporate environmental. responsibility in China. J. Clean. Prod..

[71] Li Z. (2017). The Impact of Public Participation on Local Government’s Environmental. Governance—An Analysis of Provincial Data 2003–2013. Chin. Public Adm..

[72] Egan M. (2015). Driving Water Management Change Where Economic Incentive is Limited. J. Bus. Ethics.

[73] Lambooy T. (2011). Corporate social responsibility: Sustainable water use. J. Clean. Prod..

[74] Petts G., Gurnell A. (2022). Hydrogeomorphic Effects of Reservoirs, Dams, and Diversions. Treatise Geomorphol..

[75] Qu X., Chen Y., Liu H., Xia W., Lu Y., Gang D.-D., Lin L.-S. (2020). A holistic assessment. of water quality condition and spatiotemporal patterns in impounded lakes along the eastern route of China’s South-to-North water diversion project. Water Res..

[76] Wang L., Zhang J., Shu Z., Lau C., Zhou X., Wang G. (2022). Study on the Spatio-temporal. Differentiation Characteristics of Vulnerability of Water Resources System in Henan Province. J. North China Univ. Water Resour. Electr. Power.

[77] Liu Q., Chen Y. (2016). Assessing the Vulnerability of Basin Water Resources Based. on Entropy Weight Method: A Case Study of the Huaihe River basin. J. Yangtze River Sci. Res..

[78] Su X., Li X., Liu J., Zeng F. (2018). Vulnerability assessment of water resources in. the northwest typical area based on comprehensive weighting method. J. Arid. Land Resour. Environ..

[79] Han X., Wang P., Wang J., Qiao M., Zhao X. (2020). Evaluation of human-environment. system vulnerability for sustainable development in the Liupan mountainous region of Ningxia, China. Environ. Dev..

[80] Qiu W., Zhao Q.L., Li S., Chang C.C. (2008). Ecological Security Evaluation of Heilongjiang. Province with Pressure-State-Response Model. Environ. Sci..

[81] Cao X., Hu C., Qi W., Zheng H., Shan B., Zhao Y., Qu J. (2019). Strategies for Water Resources Regulation. and Water Environment Protection in Beijing-Tianjin-Hebei Region. Strateg. Study Chin. Acad. Eng..

[82] Wooldridge J.M. (2010). Econometric Analysis of Cross Section and Panel Data.

[83] Burritt R.L., Christ K.L., Omori A. (2016). Drivers of corporate water-related disclosure: Evidence from Japan. J. Clean. Prod..

[84] Bulcke P., Vionnet S., Vousvouras C., Weder G. (2020). Nestlé’s corporate water strategy over time: A backward- and forward-looking view. Int. J. Water Resour. Dev..

[85] Callaghan P., Adapa L.M., Buisman C. (2020). How can innovation theories be applied to water technology innovation?. J. Clean. Prod..

[86] Lim K.Y., Morris D. (2022). Thresholds in natural resource rents and state owned enterprise. profitability: Cross country evidence. Energy Econ..

[87] Huang L., Liu S., Han Y., Peng K. (2020). The nature of state-owned enterprises and collection. of pollutant discharge fees: A study based on Chinese industrial enterprises. J. Clean. Prod..

[88] Matuszak P., Kabaciński B. (2021). Non-commercial goals and financial performance of state-owned enterprises—Some evidence from the electricity sector in the EU countries. J. Comp. Econ..

[89] Christ K.L. (2014). Water management accounting and the wine supply chain: Empirical evidence from Australia. Br. Account. Rev..

[90] Zhou Q., Wang Y., Zeng M., Jin Y., Zeng H. (2021). Does China’s river chief policy improve corporate water disclosure? A quasi-natural experimental. J. Clean. Prod..

[91] Tingey-Holyoak J. (2014). Sustainable water storage by agricultural businesses: Strategic responses to institutional pressures. J. Bus. Res..

[92] Zhang P., Wu H. (2022). Does the Exit Audit of Natural Resource Assets of Leading Cadres Promote the Fulfillment of Corporate Environmental Responsibility?. J. Audit. Econ..

[93] Bo C., Cunjian Y. (2021). Comparison and Reference of Water Accounting between China and Australia under the Background of Water Governance Reform. Reform Econ. Syst..

[94] Zhou Z., Zhou H., Zeng H., Chen X. (2018). The impact of water information disclosure on. the cost of capital: An empirical study of China’s capital market. Corp. Soc. Responsib. Environ. Manag..

